# Comprasion of ICare Rebound Tonometer and Goldmann Applanation Tonometer in High Myopia

**DOI:** 10.1155/2014/869460

**Published:** 2014-10-14

**Authors:** Halil Huseyin Cagatay, Metin Ekinci, Zeliha Yazar, Gokcen Gokce, Erdinc Ceylan

**Affiliations:** ^1^Department of Ophthalmology, Faculty of Medicine, Kafkas University, 36100 Kars, Turkey; ^2^Department of Ophthalmology, Sarıkamış Military Hospital, 36520 Kars, Turkey; ^3^Department of Ophthalmology, Erzurum Training and Research Hospital, 34360 Erzurum, Turkey

## Abstract

*Purpose.* To compare intraocular pressure (IOP) measurements with the Goldmann applanation tonometer (GAT) and the ICare rebound tonometer (RBT) in high myopic eyes. *Patients and Methods*. This randomized prospective study included 40 eyes of 40 patients with high myopia. All patients' central corneal thickness (CCT), anterior chamber depth (ACD), axial length (AXL), keratometry, and refractive measurements were recorded and followed by IOP measurement with RBT and GAT. *Results*. The average CCT, AXL, and ACD were determined to be 514.65 ± 32 *μ*m, 27.65 ± 2.22 mm, and 3.25 ± 0.51 mm, respectively. Mean *K* was 43.27 ± 1.4 D and mean spherical equivalent was −11.31 ± 4.30 D. The mean IOP values obtained by RBT and GAT were 17.18 ± 3.72 mmHg and 16.48 ± 3.19 mmHg, respectively. The deviations of RBT readings from corrected GAT values were highly correlated with CCT values (*r* = 0.588, *P* = 0.0001). The mean corrected GAT reading was 17.49 ± 3.01 mmHg. Linear regression analysis showed that a CCT change of 10 *μ*m resulted in an RBT reading deviation of 0.57 mmHg. The Bland-Altman scatter-plot and McNemar test showed a clinically good level of agreement between the two tonometers. *Conclusion*. This study found a good agreement level between the two tonometers in high myopic patients and that RBT measurements are influenced by CCT variations.

## 1. Introduction

Elevated intraocular pressure (IOP) is the most important risk factor in glaucoma; lowering of IOP may stop or delay the progression of disease [1, 2]. Goldmann applanation tonometer is widely accepted current gold standard for IOP measurement. This device has some disadvantages such as being affected by central corneal thickness (CCT), may cause local trauma to the corneal surface, may cross-contaminate and requires slit lamp biomicroscopy and topical anesthesia. Further, topical anesthesia may cause reflex blepharospasm and allergic reactions [3]. Therefore to eliminate these disadvantages, various alternative instruments have been developed [4–7]. The rebound tonometer (RBT) seems to be a good alternative, with advantages over other tonometers such as simple usage so that establishing cooperation with the patient is easy, quickly performed, highly reproducible, and portable and does not require anesthetic drops and slit lamp biomicroscopy [8–11]. After Kantiola's encouraging results with this tonometer, commercial production was started in 2003 under the name “ICare rebound tonometer” (ICare TA01; Tiolat, Helsinki, Finland) and it has entered our daily clinical practice in gradually increasing rates until the present day [8, 9].

The ICare rebound tonometer, which is based on the induction rebound or impact principle, monitors the motion parameters of the probe colliding with an eye. The return rate of the probe after it touches the cornea gives information about IOP; if the return is slow, low IOP values are obtained and if it is fast, high IOP values are obtained [8].

It has been established that high myopia is a risk factor in the development and progression of glaucoma [12, 13]. Marcus et al., in their review and meta-analysis involving 48161 patients, have reported that the risk of development of open-angle glaucoma increases for high myopic patients [13]. To the best of our knowledge, comparative studies on the measurement of IOP with RBT in high myopic eyes are limited [14]. The purpose of our study was to compare IOP readings taken with GAT and RBT and to assess the influence of CCT, AXL (axial length), and ACD (anterior chamber depth) on IOP measurements.

## 2. Patients and Methods

The study was conducted in accordance with the recommendations of the Declaration of Helsinki. The local medical ethics committee approved the study and informed consent for participation was obtained from each subject. This prospective and randomized study included adult patients who had no ocular pathology other than having myopia of 6 diopters or over. Exclusion criteria included presence of any ocular pathology other than high myopia and ocular hypertension.

All patients underwent a complete ophthalmologic examination, including best-corrected visual acuity evaluation, slit-lamp examination, gonioscopy, and fundus biomicroscopy with a 90-diopter lens.

The central corneal thicknesses (CCT) of all patients were measured with a central ultrasonic pachymeter (Pacline pachymeter, Optikon 2000, Italy). After the pachymeter probe was placed on the corneal center, 3 measurements were taken and the mean value was used for further analysis. The anterior chamber depth (ACD) and axial length (AXL) measurements were taken 3 times with ultrasound biometry (Bioline, Optikon 2000) and the mean value was used for further analysis. Keratometry and refractive measurements were taken with a noncontact autorefractometer (RK-F1; Canon, Tokyo, Japan).

IOP measurements with RBT and GAT were taken by two different physicians (Halil Huseyin Cagatay and Metin Ekinci), with a minimum 15-minute time interval between readings. All instruments were calibrated and measurements were performed according to instructions and guidelines of the manufacturer. The RBT software is preprogrammed for 6 measurements. After the sixth measurement, the letter P appears in the display, followed by a mean reading with a standard deviation on the screen. The RBT software discards the highest and lowest IOP readings automatically and calculates the mean IOP value from the rest. We discarded the results when the error sign appeared on the screen. After every measurement, the probe on the device was changed. After instillation of topical proparacaine anesthesia (Alcaine, Alcon, ABD), a fluorescein strip was applied and IOP measurements were taken for 3 times with the GAT (Haag Streit, Koeniz, Switzerland) and the average IOP value was determined. GAT measurements were taken by different physicians, who were blinded to the IOP measurement obtained by the RBT.

In the corrected IOP calculation, depending on the CCT, the Doughty and Zaman formula was used as follows: corrected IOP = IOP measured with  GAT − ((CCT − 535) × (2.5/50)) [15]. Corrected IOP has been accepted as the gold standard. In all patients, the difference between IOP values and RBT values was compared and analyzed. The relation of these values with CCT, ACD, AXL, keratometer parameters, and refractive measurements were examined.

## 3. Statistical Analysis

SPSS 19.0 for Windows was used for statistical analysis. Mean, standard deviation, and minimum and maximum values were calculated for continuous variables. The Shapiro-Wilk test was used for normality tests. The McNemar test was used to assess the correspondence between the GAT and RBT measurements. IOP measurements taken by the RBT were corrected according to CCT and estimated using simple linear regression analysis. The bias and 95% confidence interval of the difference between IOP measurements taken by applanation tonometry and RBT were calculated using the Bland-Altman method. The significance of the difference in terms of averages of IOP measurements between the GAT and RBT was evaluated with dependent *t*-tests. Linear regression analysis was used to evaluate the influence of CCT and the mean IOP value of the tonometers and the differences between these two methods. The cases were evaluated unilaterally, with the measurements taken only from their right eyes. A  *P*  value of <0.05 was considered statistically significant. The percentage of eyes with an IOP difference between tonometers within ±1, ±2, and ±3 was calculated. The correlation between tonometers and the influence of ACD, AXL, CCT, refractive errors, and keratometry values was evaluated using Spearman's Rho correlation coefficients. A power calculation using G-Power (verison 3.1.3) was performed to calculate effect size and power of study.

## 4. Results

This study included 40 right eyes of 40 high myopic subjects, having a mean age of 35.73 ± 12.97 years (range, 18–63 years). After the measurements were taken by RBT and GAT, the mean of IOP values was 17.18 ± 3.72 mmHg and  16.48 ± 3.19 mmHg, respectively. The mean corrected GAT reading, calculated according to the Doughty and Zaman formula, was 17.49 ± 3.01 mmHg [15] (Table 1). The average CCT, AXL, and ACD were determined to be 514.65 ± 32 *μ*m, 27.65 ± 2.22 mm, and 3.25 ± 0.51 mm, respectively. Refractive errors and keratometry values were shown in Table 2.

The correspondence between GAT and RBT measurement results was investigated using the McNemar test; there was no significant difference between two measurements (*P* = 1.000), indicating that the RBT and GAT results were in good agreement.

**Figure 1:**
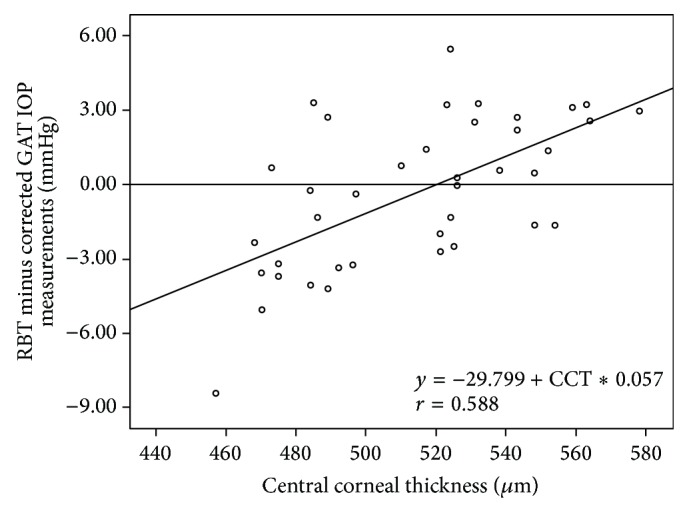
Correlation between CCT and the deviation of the RBT measurements from the corrected GAT values calculated according to the formula derived from the studies of Doughty and Zaman [15].

The deviations of the RBT from the corrected GAT values were correlated with the CCT values (*r* = 0.588, *P* = 0.0001) (Figure 1). Because the linear regression function was *y* = −29.799 + CCT∗0.057 and the linear regression line intercepted the *x*-axis at the CCT value of 519.9 mm (Figure 1), the following correction formula for the RBT was used: corrected RBT IOP = measured RBT  IOP − (CCT − 519.9) × 0.057. According to this formula, every change of 10 *μ* in CCT level caused a 0.57 mmHg change in IOP (*P* < 0.001).

There was no correlation of IOP measurements by either instrument with AXL (*P* = 0.899) and with ACD (*P* = 0.166) in myopic patients. There was no correlation with IOP measurements taken by each instrument with *K*1 (*P* = 0.070), *K*2 (*P* = 0.440), mean *K* (*P* = 0.171), spherical value (*P* = 0.239), cylindrical value (*P* = 0.860) and spherical equivalent values (*P* = 0.233).

**Figure 2:**
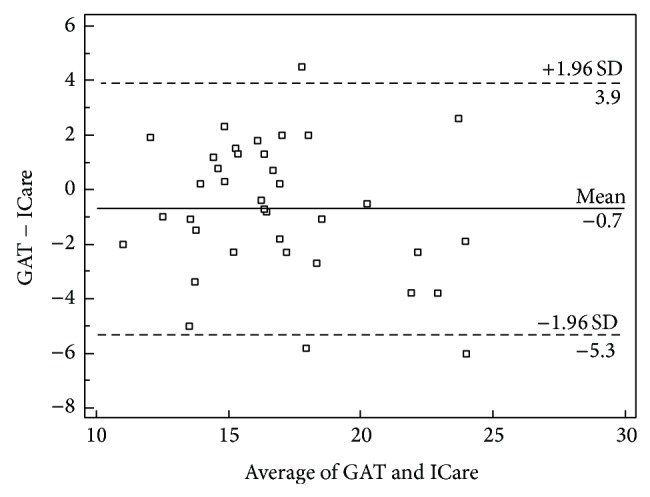
Bland-Altman analysis showing the distribution of differences in IOP (GAT tonometer value minus RBT value, mmHg) (*y*-axis) and the mean IOP value of the tonometers (*x*-axis) for each eye measured.

The Bland-Altman scatter-plot comparing the GAT and RBT readings (Figure 2) showed good agreement between the 2 methods. The differences between corresponding measures (RBT value minus GAT value) had a mean of 0.71 mmHg, a standard deviation of 2.35 mmHg, and a 95% confidence interval of −5.3 to 3.9 mmHg. These differences appeared to be nonsignificant, as shown in the plot (*P* = 0.097) in Figure 2.

According to the RBT readings of IOP, 30.0% of the subjects were within ±1 mmHg of IOP performed with the GAT, 67.5% of the subjects were within ±2 mmHg, and 82.5% of the subjects were within ±3 mmHg. ICare rebound tonometer identified an IOP of 21 mmHg or above in 5 of 6 subjects with a GAT identified IOP equal to or above 21 mmHg (RBT has a sensitivity of 83.3%). As well, RBT identified an IOP under 21 mmHg in 33 of 34 subjects having a GAT IOP under 21 mmHg (RBT has a specificity of 97.1%). Power of this study was 0.85 and effect size was 0.40.

## 5. Discussion

Accurate and reliable measurement of intraocular pressure is extremely important in the diagnosis and follow-up of the glaucoma. Various measurement methods have been used previously in the determination of IOP [4]. In previous studies, it has been shown that the RBT gives higher results than the GAT, especially in adult patients, and it has been mentioned by many authors that there is a reasonable agreement between the RBT and the GAT, which is accepted as the gold standard in IOP measurement for various patient groups [16–23]. Also, a medium or high level of correlation between the GAT and the RBT results has been reported. Previous studies were conducted with pathologic corneas, healthy eyes, pediatric, or glaucoma subjects and there is limited literature on high myopic patients.

Our study aimed to determine the reliability of the RBT in patients with myopia higher than 6 D, its correlation level with the GAT, and the influence of CCT, AXL, and ACD on the results for each measurement method. Chui et al. reported no significant difference in measurements made from the central and temporal/peripheral cornea depending on CCT and there was no correlation between RBT measurements and CCT values [24]. In our study, in eyes having myopia of 6 dioptry or over the good level of clinical agreement that has been determined with the GAT was independent of the RBT's axial lengths.

In our study, in accordance with the literature, the RBT measurements were affected by CCT and every 10 *μ*m change in CCT level caused a 0.57 mmHg change in the intraocular pressure obtained with RBT (*P* < 0.001) [25, 26]. In addition to the influence of CCT on IOP measurements obtained with the RBT, this tonometer has been shown not to be significantly correlated with ACD, AXL, keratometry, and refractive values.

Limitations of this study were the relatively small number of eyes for IOP analysis and did not measure the hysteresis of the participant corneas.

To the best of our knowledge, comparative studies on the measurement of IOP with RBT in high myopic eyes are limited. Avitabile et al. in their study evaluated the refractive errors on IOP measurements with Icare and Goldmann applanation tonometry [14]. Although they did not classify the level of the myopia, they reported that in myopic eyes, the RT-GAT difference is correlated with the refraction, but not with the CCT. In controversy of this finding, we did not obtain a correlation with refraction and RT-GAT difference in high myopic eyes.

In our study, there was no significant difference between average IOP values obtained with the RBT and the GAT in high myopic eyes. It was also determined that the RBT measurements were affected by CCT and there was a clinically acceptable correlation between the two devices as determined by linear regression analysis and Bland-Altman analysis. We suggest that the RBT can be used in IOP measurement by being combined with pachymeter measurements in high myopia patients. Studies involving more patients or intraocular manometric measurements are needed to determine the reliability of the RBT in high myopic patients.

## Figures and Tables

**Table 1:** IOP measurement results.

	RBT readings (mmHg)	GAT readings (mmHg)	CORRECTED GAT readings (mmHg)	GAT-ICARE (mmHg)
Mean	17.18	16.48	17.49	−0.71
Standard deviation	3.72	3.19	3.01	2.35
Range	11.10–27.00	10–25	11.25–24.05	−4.50–6.00

**Table 2:** Refractive errors and keratometry values.

	*K*1 (D)	*K*2 (D)	Mean *K* (D)	Spherical value (D)	Cyclindiri*c* value (D)	Spherical equivalent (D)
Mean	42.46	44.08	43.27	−10.44	−1.75	−11.31
Standard deviation	1.48	1.5	1.4	4.28	0.96	4.30
Range	40.25–45.75	10–25	11.25–24.05	−6.0–−23.0	−0.25–3.75	−6.38–24.50

## References

[1] Landers J., Goldberg I., Graham S. L. (2002). Analysis of risk factors that may be associated with progression from ocular hypertension to primary open angle glaucoma. *Clinical and Experimental Ophthalmology*.

[2] Kass M. A., Heuer D. K., Higginbotham E. J., Johnson C. A., Keltner J. L., Philip Miller J., Parrish R. K., Roy Wilson M., Gordon M. O. (2002). The Ocular Hypertension Treatment Study: a randomized trial determines that topical ocular hypotensive medication delays or prevents the onset of primary open-angle glaucoma. *Archives of Ophthalmology*.

[3] Whitacre M. M., Stein R. (1993). Sources of error with use of Goldmann-type tonometers. *Survey of Ophthalmology*.

[4] ElMallah M. K., Asrani S. G. (2008). New ways to measure intraocular pressure. *Current Opinion in Ophthalmology*.

[5] Minckler D. S., Baerveldt F., Heuer D. K. (1987). Clinical evaluation of the Oculab Tonopen. *American Journal of Ophthalmology*.

[6] Koçak I., Orgül S., Saruhan A., Haefliger I., Hendrickson P., Flammer J. (1998). Measurement of intraocular pressure with a modern noncontact tonometer. *Ophthalmologica*.

[7] Tonnu P.-A., Ho T., Newson T., El Sheikh A., Sharma K., White E., Bunce C., Garway-Heath D. F. (2005). The influence of central corneal thickness and age on intraocular pressure measured by pneumotonometry, non-contact tonometry, the Tono-Pen XL, and Goldmann applanation tonometry. *The British Journal of Ophthalmology*.

[8] Kontiola A. I. (2000). A new induction-based impact method for measuring intraocular pressure. *Acta Ophthalmologica Scandinavica*.

[9] Kontiola A. I. (2003). *Development impact tonometers for clinical use and glaucoma research [Academic Dissertation]*.

[10] Pakrou N., Gray T., Mills R., Landers J., Craig J. (2008). Clinical comparison of the Icare tonometer and goldmann applanation tonometry. *Journal of Glaucoma*.

[11] Kontiola A., Puska P. (2004). Measuring intraocular pressure with the Pulsair 3000 and Rebound tonometers in elderly patients without an anesthetic. *Graefe's Archive for Clinical and Experimental Ophthalmology*.

[12] Xu L., Wang Y., Wang S., Jonas J. B. (2007). High myopia and glaucoma susceptibility. The Beijing Eye study. *Ophthalmology*.

[13] Marcus M. W., de Vries M. M., Junoy Montolio F. G., Jansonius N. M. (2011). Myopia as a risk factor for open-angle glaucoma: a systematic review and meta-analysis. *Ophthalmology*.

[14] Avitabile T., Longo A., Rocca D., Amato R., Gagliano C., Castaing M. (2010). The influence of refractive errors on IOP measurement by rebound tonometry (ICare) and Goldmann applanation tonometry. *Graefe's Archive for Clinical and Experimental Ophthalmology*.

[15] Doughty M. J., Zaman M. L. (2000). Human corneal thickness and its impact on intraocular pressure measures: a review and meta-analysis approach. *Survey of Ophthalmology*.

[16] Brusini P., Salvetat M. L., Zeppieri M., Tosoni C., Parisi L. (2006). Comparison of ICare tonometer with Goldmann applanation tonometer in glaucoma patients. *Journal of Glaucoma*.

[17] García-ResúA C., González-Meijome J. M., Gilino J., Yebra-Pimentel E. (2006). Accuracy of the new ICare rebound tonometer vs. other portable tonometers in healthy eyes. *Optometry & Vision Science*.

[18] Flemmons M. S., Hsiao Y. C., Dzau J. (2011). Icare rebound tonometry in children with known and suspected glaucoma. *Journal of American Association for Pediatric Ophthalmology and Strabismus*.

[19] Marini M., da Pozzo S., Accardo A., Canziani T. (2011). Comparing applanation tonometry and rebound tonometry in glaucomatous and ocular hypertensive eyes. *European Journal of Ophthalmology*.

[20] Kageyama M., Hirooka K., Baba T., Shiraga F. (2011). Comparison of ICare rebound tonometer with noncontact tonometer in healthy children. *Journal of Glaucoma*.

[21] Rosentreter A., Athanasopoulos A., Schild A. M., Lappas A., Cursiefen C., Dietlein T. S. (2013). Rebound, applanation, and dynamic contour tonometry in pathologic corneas. *Cornea*.

[22] Sahin A., Basmak H., Yildirim N. (2008). The influence of central corneal thickness and corneal curvature on intraocular pressure measured by tono-pen and rebound tonometer in children. *Journal of Glaucoma*.

[23] Köktekir Ekinci B., Bakbak B., Gedik Ş. (2012). Comparison of intraocular pressure measured with rebound tonometer, pneumotonometer and goldmann applanation tonometer in healthy individuals. *Turkiye Klinikleri Journal of Ophthalmology*.

[24] Chui W.-S., Lam A., Chen D., Chiu R. (2008). The influence of corneal properties on rebound tonometry. *Ophthalmology*.

[25] Rao A., Kumar M., Prakash B., Varshney G. (2014). Relationship of central corneal thickness and intraocular pressure by iCare rebound tonometer. *Journal of Glaucoma*.

[26] Salim S., Du H., Wan J. (2013). Comparison of intraocular pressure measurements and assessment of intraobserver and interobserver reproducibility the and Goldmann Applanation Tonometer in glaucoma patients. *Journal of Glaucoma*.

